# Using Blood Gas Analysis and Capnography to Determine Oxygenation Status in Bottlenose Dolphins (*Tursiops truncatus*) Following the *Deepwater Horizon* Oil Spill

**DOI:** 10.3390/toxics11050423

**Published:** 2023-05-03

**Authors:** Sarah M. Sharp, Forrest M. Gomez, Jenny M. Meegan, Teresa K. Rowles, Forrest Townsend, Lori H. Schwacke, Cynthia R. Smith

**Affiliations:** 1 National Marine Mammal Foundation, San Diego, CA 92106, USA; 2 International Fund for Animal Welfare, Yarmouth Port, MA 02675, USA; 3 Marine Mammal Health and Stranding Response Program, National Oceanographic and Atmospheric Administration, Silver Spring, MD 20910, USA; 4 College of Veterinary Medicine, Auburn University, Auburn, AL 36832, USA

**Keywords:** dolphin, oil spill, respiratory disease, blood gas, oxygenation, capnography, pregnancy

## Abstract

Following the *Deepwater Horizon* (DWH) oil spill in 2010, poor pulmonary health and reproductive failure in bottlenose dolphins (*Tursiops truncatus*) in the northern Gulf of Mexico were well-documented. One postulated etiology for the increased fetal distress syndrome and pneumonia found in affected perinatal dolphins was maternal hypoxia caused by lung disease. The objective of this study was to evaluate the utility of blood gas analysis and capnography in determining oxygenation status in bottlenose dolphins with and without pulmonary disease. Blood and breath samples were collected from 59 free-ranging dolphins in Barataria Bay, Louisiana (BB), during a capture–release health assessment program, and from 30 managed dolphins from the U.S. Navy Marine Mammal Program in San Diego, CA. The former was the oil-exposed cohort and the latter served as a control cohort with known health histories. Capnography and select blood gas parameters were compared based on the following factors: cohort, sex, age/length class, reproductive status, and severity of pulmonary disease. Animals with moderate–severe lung disease had higher bicarbonate concentrations (*p* = 0.005), pH (*p* < 0.001), TCO_2_ (*p* = 0.012), and more positive base excess (*p* = 0.001) than animals with normal–mild disease. Capnography (ETCO_2_) was found to have a weak positive correlation with blood PCO_2_ (*p* = 0.020), with a mean difference of 5.02 mmHg (*p* < 0.001). Based on these findings, indirect oxygenation measures, including TCO_2_, bicarbonate, and pH, show promise in establishing the oxygenation status in dolphins with and without pulmonary disease.

## 1. Introduction

The *Deepwater Horizon* oil spill (DWH), one of the largest environmental disasters in US history, leaked nearly 3.2 million barrels of crude oil into the Gulf of Mexico in 2010 [1]. In the aftermath of the spill, poor pulmonary health and reproductive failure were well-documented conditions for inshore bottlenose dolphins, *Tursiops truncatus*, in the northern Gulf of Mexico [2,3,4,5,6] Lung ultrasound examinations conducted during live dolphin health assessments showed pulmonary nodules, masses, consolidation, and pleural effusion [2,6]. These lesions were consistent with pneumonia observed during necropsies of dolphins found stranded during the same time period [4]. Vessel-based surveys revealed low reproductive success of bottlenose dolphins in the northern Gulf of Mexico, with only 19.4% of pregnant females producing viable calves, compared to 64.7% in populations not impacted by the spill [5]. Simultaneously, perinatal dolphin strandings in the region also increased, and these young animals were more likely to have fetal distress syndrome and non-lungworm-associated pneumonia than perinates from a non-oiled population [7].

The mechanism of pulmonary dysfunction due to exposure to oil and oil dispersants is unknown in dolphins, but one in vitro study on human airway epithelial cells suggested that key molecular signatures coinciding with common lung diseases (upregulation of gene sets involved in angiogenesis and immune response and downregulation of those involved in cell junctions and steroid synthesis) were present in exposed cells [8]. Another study in rats found that oil exposure resulted in severe airway inflammation, changes in protein profiles and serum metabolism, and disturbances in microbial community structure [9]. In Barataria Bay dolphins, there may also be a link between pulmonary disease and reproductive failure. One proposed etiology for the increase in fetal distress and perinatal death was maternal hypoxia due to concurrent lung disease in the mothers. Various manifestations of lung disease pose a significant barrier to blood oxygenation including decreased ventilatory drive, intra-alveolar exudates, damage to alveolar capillaries, airway obstruction, and septal thickening by edema, inflammation, and/or fibrosis [10]. The human literature suggests that persistent maternal hypoxia may cause significant injury to vital organs, including the placenta, which may in turn have profound effects on the developing fetus. Documented fetal impacts include intrauterine growth restriction (IUGR), asphyxia, multiorgan failure, premature delivery, and perinatal demise [11]. Additionally, premature rupture of membranes (amniotic sac) in humans has been associated with maternal respiratory diseases such as pneumonia and bronchitis [12].

Current methods for evaluating oxygenation status include blood gas analysis, capnography, and pulse oximetry. Arterial blood gas is the gold standard for evaluating oxygenation and ventilatory status in human and veterinary patients [13,14]. Oxygen (O_2_) and carbon dioxide (CO_2_) are the most important respiratory gases, and their partial pressures in arterial blood reflect the effectiveness of the body’s gas-exchange system. pH is a measurement of hydrogen ion activity in the blood that indicates how acidic or basic the patient’s blood is. Bicarbonate (HCO_3_), anion gap, and base excess (BE) are calculated values that help qualify acid–base disturbances [15]. Blood gas values have been used to investigate dolphin physiology [16,17] and are used in clinical practice to evaluate the acid–base status in managed dolphin populations, especially during anesthesia [18]. Obtaining a purely arterial sample peripherally from dolphins is difficult due to the presence of periarterial venous rete that result in a mixture of venous and arterial blood. However, several blood gas parameters (PCO_2_, PO_2_, and pH) recorded from the central artery of the fluke’s periarterial venous rete correlated well with those taken from the carotid artery during anesthesia [19,20].

Capnography is a non-invasive diagnostic tool that provides clinically pertinent information regarding a patient’s ventilatory status. In mammals, end-tidal partial pressures of carbon dioxide (ETCO_2_) are typically 5 mmHg lower than the corresponding arterial partial pressures of carbon dioxide (PaCO_2_) due to physiologic dead space diluting the CO_2_ present in the exhaled gases. Considered the standard of care for monitoring intubated animals under anesthesia [21], ETCO_2_ has proven clinically useful in monitoring anesthetized dolphins [19,22]. Additionally, Kelmer et al. [23] found nasal capnography to be an effective method for estimating PaCO_2_ in critically ill, awake dogs. In awake dolphins, ETCO_2_ values have been found to be comparable to those of terrestrial mammals [24] and ETCO_2_ has been shown to increase in a dolphin with pneumonia [25], but otherwise, its clinical utility in dolphins has not been evaluated in the literature.

Pulse oximetry is another useful non-invasive tool that provides important information regarding patient peripheral blood oxygenation and perfusion. Pulse oximeter probes use light to indirectly measure hemoglobin saturation in peripheral arterial blood (SpO_2_%) through non-pigmented skin or mucous membranes. The SpO_2_ is not always identical to the SO_2_ measured by arterial blood gas analysis, but the two are sufficiently correlated for clinical utility. Normally, SpO_2_ is 95–99% in healthy humans and animal species for which there are sufficient data. Pulse oximetry has been successfully measured using a probe on the tongue of anesthetized dolphins (Linnehan and MacMillan 1991), but little success has been reported using reflectance probes in awake or sedated dolphins rectally or within the genital slit [22]. Pulse oximetry on the tongue in awake managed dolphins has also been attempted, but it is difficult to keep sufficiently stationary to avoid motion artifacts.

The objective of this study was to evaluate the utility of blood gas analysis, capnography, and pulse oximetry in determining oxygenation status in bottlenose dolphins with and without pulmonary disease. Samples were collected from both wild-caught bottlenose dolphins in an area affected by DWH and a control managed population of conspecifics in San Diego, CA, with known medical histories. We hypothesized that animals with pulmonary disease would have lower pH, PO_2_, and SpO_2_ and higher ETCO_2_, PCO_2_, HCO_3_, and lactate than healthy animals.

## 2. Materials and Methods

### 2.1. Field-Based Dolphin Sample Collection

As part of health assessment studies following the DWH oil spill, samples were collected in Barataria Bay, Louisiana (BB), during three time periods: 11–22 July 2016, 14–21 June 2017, and 18–21 September 2017. Bottlenose dolphins were temporarily caught, sampled, and then released onsite following procedures that were reviewed and approved by the National Oceanographic and Atmospheric Administration’s Institutional Animal Care and Use Committee. The capture methods have previously been described [26], as have the general health assessment procedures [2,6,27]. Outward signs of pulmonary disease noted upon physical exam included abnormal pulmonary auscultation (such as rales, wheezes, decreased or absent lung sounds), malodorous blow, discolored blowhole mucous, or plaques. BB dolphins were stratified by the following length classes: juvenile (≤225 cm), subadult (>225 cm <240 cm), and adult dolphins (≥240 cm) [28].

Full-body ultrasound examinations were conducted, including pulmonary ultrasound, as described by Smith et al. [29] and Schwacke et al. [2]. Separate lung scores (normal, mild, moderate, severe) for each lung were assigned by two experienced marine mammal veterinarians (F.G. and C.S.) based on overall findings and the presence/absence and severity of abnormalities. Only dolphins with bilateral lung scores were used for this analysis. Overall lung scores were assigned based on the most severe unilateral lung score (i.e., overall score was ‘moderate’ if one or more lungs had a maximum score of ‘moderate’).

In addition to the blood collection described in the above publications, samples for blood gas analysis were obtained from the ventral fluke vasculature with a PICO50 aspirator syringe (Radiometer, Brea, CA, USA). Majority of samples were run within 20 min of collection on CG4+ and CHEM8+ cartridges on an i-STAT 1 System handheld analyzer (Abbott Point of Care, Princeton, NJ, USA) in a climate-controlled shipboard lab. The i-STAT cartridges each have chemically sensitive biosensors on a silicon chip that is configured to run tests, specifically: base excess (BEecf), bicarbonate (HCO_3_), partial pressure of carbon dioxide (PCO_2_), partial pressure of oxygen (PO_2_), oxygen saturation (sO_2_), total carbon dioxide (TCO_2_), pH, and lactate on the CG4+, and anion gap, chloride, creatinine, glucose, hematocrit (Hct), hemoglobin, ionized calcium, potassium, sodium, blood urea nitrogen (BUN), and TCO_2_ on the CHEM8+. The time between the net set and the blood gas sample collection was noted for each case as an indicator of the potential exertion duration.

**Figure 1:**
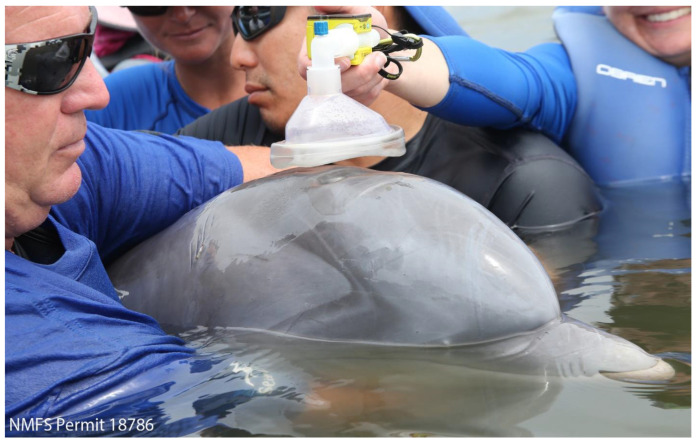
Capnography sampling of wild-caught *T. truncatus* in Barataria Bay, LA.

All animals were gently restrained in the water for capnography (Figure 1). Five sequential ETCO_2_ values were collected with an EMMA portable capnograph (Masimo Corporation, Irvine, CA) using a standard rubber surgical mask held immediately over the blowhole (within 2 cm) attached to the EMMA adapter. The stand-off distance allowed the animal to breathe normally during data collection, which mitigated the risk of anxiety-related changes in respiratory rate or depth that could occur when placing pressure around the blowhole. Capnography data collection was synchronized with blood gas sampling at both the start (pre) and the end (post) of the health assessment procedures. The mean of each of these five ETCO_2_ samples per individual was used in the analysis. Unless otherwise indicated, all values used for analysis (both capnography and blood gas) were values obtained at the beginning of the procedures (pre). No capnograms were evaluated as part of this study.

Prognostic scores were established for all BB dolphins by two or more experienced marine mammal veterinarians (F.G. and C.S.) based on all available data for each animal, including: heart rate, respiratory rate, and respiratory character; attitude, responsiveness, and overall stability; subjective body condition score and objective body mass index; complete blood counts and serum chemistries; fecal and blowhole cytology, when available; sonograms, specifically pulmonary, renal, and reproductive; oral exams; skin evaluations, and age analyses. Assigned prognostic scores from best to worst were good, fair, guarded, poor, and grave.

Reproductive success of pregnant BB females identified by ultrasound examination during the health assessments was evaluated using vessel-based resight surveys. Previously captured pregnant individuals were identified through photo analysis using a combination of freeze brands, tag placement, and/or fin notches [3]. An estimated due date for each pregnant female was calculated based on the biparietal skull diameter upon ultrasound examination using previously described methods [30]. Following the estimated due date, presence of an associated calf with each pregnant individual was assessed via photographs. If a calf was sighted swimming alongside its presumed mother, the outcome was classified as a success. If a pregnant female was observed two weeks to one year following its due date without an associated neonate/calf, the outcome was classified as a reproductive failure (due either to fetal or neonatal death).

### 2.2. Managed Dolphin Sample Collection

Between 1 August 2016 and 12 January 2017, bottlenose dolphins cared for by the U.S. Navy Marine Mammal Program in San Diego, CA, USA, were sampled as part of their routine care and health monitoring plans, as directed by their attending veterinarian. The Secretary of Navy Instruction 3900.41H directs that Navy marine mammals be provided the highest quality of care. The Navy program is accredited by AAALAC International and adheres to the national standards of the U.S. Public Health Service Policy on the Humane Care and Use of Laboratory Animals and the Animal Welfare Act.

Dolphins with underlying pulmonary disease and pregnant females were prioritized for inclusion in this study to provide a comparable sample to the wild population. Dolphins either voluntarily presented their ventral flukes while in water for blood sampling from the ventral fluke arteriovenous rete, or they were gently restrained for blood collection during routine health exams. After the clinical blood sample was drawn, an additional PICO syringe of blood was collected for blood gas analysis. CG4+ cartridges were run on the i-STAT 1 analyzer within 10 min of sample collection and CHEM8+ cartridges were run within 20 min of sampling. Capnograph data were collected voluntarily just prior to blood sampling since the animal’s blowhole was submerged during the voluntary ventral fluke blood draw. Five sequential ETCO_2_ values were collected using a methodology identical to the BB dolphins, and the mean value for each dolphin was used for analysis. For a small number of cases, dolphins were sampled when out of the water on a foam mat. In these animals, blood was drawn from the caudal peduncle vessels at the insertion of the flukes and capnography data were concurrently collected.

Pulmonary ultrasounds were conducted on all sampled animals within one month of capnography and blood gas sampling. Lung scores were assigned by the attending clinician according to the same protocol as the BB dolphin cohort, and only dolphins with bilateral scores were used for this study. Based on known ages in this cohort, samples were stratified according to the following age classes: juvenile (0–5 years), subadult (>5 and <10 years), and adult (≥10 years).

Physical examinations were conducted on all sampled animals following similar methods as in the field (evaluating responsiveness, attitude, skin condition, body condition, oral cavity, respiratory rate and character, and heart rate and character). Signs of pulmonary disease upon physical exam were the same as for wild dolphins (abnormal pulmonary auscultation such as rales, wheezes, decreased or absent lung sounds, malodorous blow, discolored blowhole mucous, or plaques).

Just prior to capnograph sampling, pulse oximetry (SpO_2_) values were collected with a Rad 57 Pulse CO-Oximeter (Masimo Corporation, Irvine, CA, USA) using a LNCS AH series TF-AH Transflectance sensor or a YI AH multisite sensor. The probe was held on a sampling site for 1–2 min until SpO_2_ values reached a plateau. Various attachment methodologies were employed, including clips, tongue depressors, varying digital pressure, and multiple sampling sites (tongue, sublingual mucous membranes, genital slit), with the aim of obtaining accurate readings and establishing a standardized protocol.

### 2.3. Data and Statistical Analysis

In order to facilitate age/length class comparisons among cohorts, corresponding length thresholds for the age categories were derived from previously published growth curves (McFee et al. 2010) from another Northern Gulf of Mexico dolphin stock (Mississippi Sound). The resulting corresponding length/age classes were as follows: juvenile (≤225 cm or 0–5 years), subadult (>225 cm <240 cm or >5 and <10 years), and adult (≥240 cm or ≥10 years). In order to compare dolphins with varying degrees of pulmonary disease severity, animals were divided into two categories based on their overall ultrasound lung scores: (1) animals with normal to mild lung scores and (2) animals with moderate to severe lung scores [6]. Of the available i-STAT data, certain parameters were selected for comparison based on their clinical value in determining the oxygenation status (PCO_2_, TCO_2_, PO_2_, pH, HCO_3_, lactate, and base excess). Robust reference intervals have not been established for bottlenose dolphin blood gas parameters or end-tidal CO_2_; therefore, the dataset was analyzed both as a whole and stratified by cohort (BB or Navy).

Data normality was analyzed with the Shapiro–Wilk test for normal distribution. For all normal data, a multivariate analysis of variance (MANOVA) was performed with the Wilk’s Lambda statistic [31]. Dependent variables included in the model were: ETCO_2_, PCO_2_, TCO_2_, PO_2_, pH, HCO_3_, lactate, and base excess. Independent variables (factors) included were: lung score severity (normal–mild or moderate–severe), cohort (Navy or BB), sex (male or female), reproductive status (pregnant or non-pregnant female), and age/length class (juvenile, subadult, or adult). Additionally, the BB cohort data were compared by year (2016 or 2017) and prognostic score (good, fair, guarded, poor, or grave), and the Navy cohort data were compared by sampling scenario (out-of-water vs. in-water sampling). If significant differences were identified, two-tailed T-tests or one-way ANOVAs were performed to identify which of the dependent variables varied according to the identified factor(s).

For all non-normally distributed data, Mann–Whitney U or Kruskal–Wallis tests were performed for each variable/factor combination. Significance for analyses was set at α < 0.05, except for multiple comparisons among non-parametric data. In these cases, the Holm’s Sequential Bonferroni correction was applied to evaluate for significance [32,33].

Paired values for PCO_2_ and ETCO_2_ were compared by means of the Pearson correlation method, and correlation coefficients were interpreted as weak (<0.4), moderate (0.4–0.7), or strong (>0.7). The level of agreement between the two assays was assessed using the Bland–Altman method [34]. The change over time (time differential) of select variables (ETCO_2_, PCO_2_, HCO_3_, and lactate) for BB dolphins with available pre- and post-test data was also investigated using a combination of MANOVA and Mann–Whitney U tests. Statistical analyses were performed using MedCalc for Windows, version 17.1 (MedCalc Software, Ostend, Belgium), with the exception of MANOVA analyses, which were performed using XLSTAT for Microsoft Excel 2010 (Addinsoft, New York, NY, USA).

## 3. Results

Samples were collected from 59 (34 female, 25 male) wild-caught dolphins in Barataria Bay, Louisiana, and 30 dolphins (16 female, 14 male) cared for by the Navy in San Diego, California. Juveniles were the largest age class sampled (*n* = 41), with adults (*n* = 31) and subadults (*n* = 17) also represented (see summary data in Table 1). All wild females and those actively involved in the Navy breeding program received a reproductive ultrasound. Fifteen of fifty (30%, 5 Navy, 10 BB) were confirmed pregnant (fetus identified on the ultrasound), with 80% (12/15) in their first trimester and the remainder in their second trimester. Of the identified pregnancies, 6 successfully resulted in calves (3 Navy, 3 BB), 6 failed (2 Navy, 4 BB), and the outcome could not be determined in 3 BB cases.

### 3.1. Pulmonary Ultrasound

For BB dolphins, 19 of 59 (32%) had moderate (*n* = 16) to severe (*n* = 3) lung scores on the ultrasound, 36 had mild lung scores, and 4 were within normal limits. Eight pregnant BB dolphins had mild lung scores (all known pregnancy failures and successes fell in this group), one had a moderate lung score (unknown pregnancy outcome), and one had a normal lung score (unknown pregnancy outcome). Five of thirty Navy dolphins had mild lung scores, with the remainder having normal pulmonary ultrasounds. All pregnant Navy dolphins had normal lung scores. One Navy dolphin had a history of chronic pulmonary disease (inactive at the time of sampling), with no lesions on pulmonary ultrasound and nothing of clinical concern reported on a CT scan during the study period (considered ‘normal’ for this analysis). See Table 2 for a summary of all pulmonary ultrasound scores.

No moderate or severe lung disease cases were identified within the Navy cohort. Due to this finding and the availability of baseline physical examination data for these animals, the within-cohort analysis for Navy dolphins was performed comparing those with (1) normal lung scores and normal physical exam findings to those with (2) mild lung scores, evidence of pulmonary disease upon physical exam, or a history of chronic pneumonia. For all other lung score comparisons (all dolphins and BB dolphins), the previously described normal–mild and moderate–severe ultrasound lung score categories were employed [6].

### 3.2. Physical Exam

Thirteen BB dolphins had outward signs of respiratory disease upon physical exam, all of which had either mild (*n* = 7), moderate (*n* = 4), or severe (*n* = 2) lung scores on the ultrasound. One of three (33%) BB dolphins with severe and twelve of sixteen (75%) with moderate lung scores had no signs of respiratory disease during the physical examination. One Navy dolphin had mildly abnormal respiratory findings upon physical exam but a normal pulmonary ultrasound score, and another had a history of chronic fungal pneumonia (no clinical signs at the time of sampling). Three of five (60%) Navy dolphins with sonographic evidence of mild pulmonary disease also showed mild outward signs of respiratory disease upon physical examination. There was no difference in the respiratory rate between dolphins with normal–mild lung scores and those with moderate–severe lung scores (*p* = 0.103).

### 3.3. Pulse Oximetry

Eighteen Navy dolphins were evaluated with pulse oximetry. Twelve (66%) had plateaued SpO_2_ values of <90%, indicating significant hypoxemia, a finding which was not consistent with the clinical presentation (pink mucous membranes) or corresponding blood gas values. Due to the doubtful clinical utility of this measurement within this context, pulse oximeter values were not included in the analysis.

### 3.4. ETCO_2_ and PCO_2_

**Figure 2:**
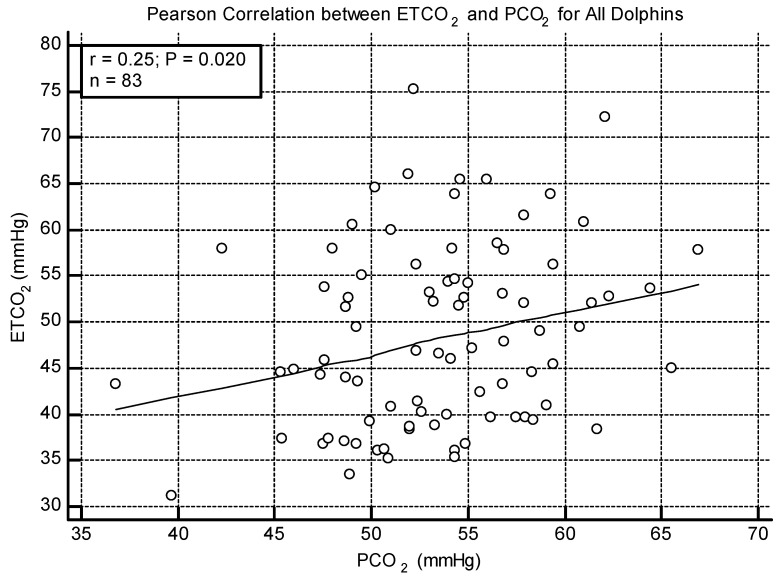
Scatterplot showing the weak positive correlation between ETCO_2_ and PCO_2_ for all dolphins.

**Figure 3:**
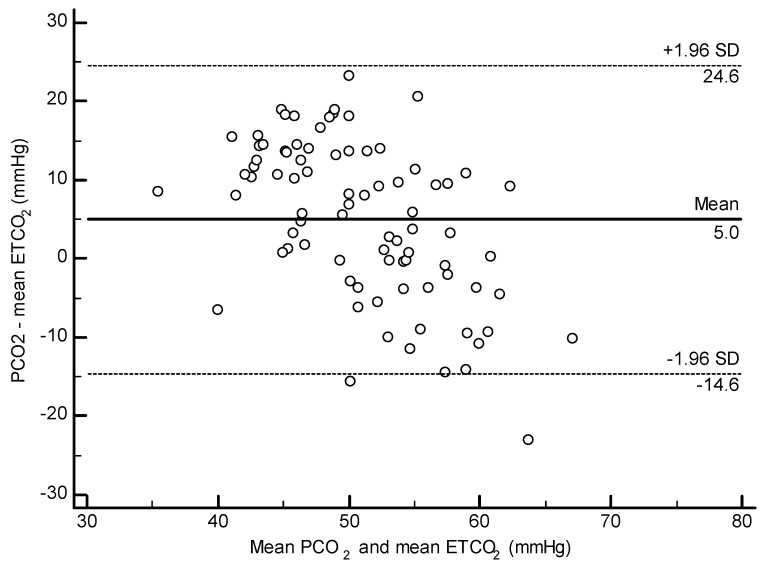
Bland–Altman plot for agreement between ETCO_2_ and PCO_2_ in all dolphins. Mean difference of 5.03 mmHg, *p* < 0.0001.

No significant correlation was found between ETCO_2_ and PCO_2_ when the animals were split by lung score category. In all other analyses (all dolphins, BB only, and Navy only), a weak positive correlation was found between ETCO_2_ and PCO_2_ (All: r = 0.25 *p* = 0.020, Figure 2; BB: r = 0.29, *p* = 0.036; Navy r = 0.38, *p* = 0.041). The Bland–Altman method was significant for all dolphins and the Navy cohort, indicating that PCO_2_ was higher than ETCO_2_ by a mean difference of 5.03 mmHg (*p* < 0.001, Figure 3) overall and 11.4 mmHg for the Navy cohort (*p* < 0.001). The Bland–Altman analysis was not significant when the BB cohort was analyzed separately.

### 3.5. All Dolphin Analysis

In dolphins with moderate–severe lung scores, HCO_3_ (*p* = 0.005), TCO_2_ (*p* = 0.027), and pH (*p* < 0.001) were higher, and base excess was more positive (*p* = 0.001), compared to dolphins with normal–mild lung scores. PO_2_ (*p* < 0.001), ETCO_2_ (*p* < 0.001), and lactate (*p* < 0.001) were higher in BB dolphins compared to Navy dolphins. The only significant difference between the sexes was a higher PCO_2_ in males than females (*p* < 0.001). No other factors significantly influenced the variables. Although non-significant, ETCO_2_ trended higher in dolphins with moderate–severe lung scores (*p* = 0.125). See Table 3 for a summary of these findings.

### 3.6. Navy Cohort

Navy dolphins sampled out of the water had higher PO_2_ than those sampled while in the water, likely due to the difference in sampling method (mean out-of-water = 66.4 mmHg, mean in-water = 44.1 mmHg, *p* < 0.001). Adult Navy dolphins had significantly lower ETCO_2_ than juveniles or subadults (juvenile median = 44.1 mmHg, subadult = 50.6 mmHg, adult = 42.8 mmHg, *p* = 0.019). No other factors significantly influenced the variables. 

### 3.7. Barataria Bay Cohort

**Figure 4:**
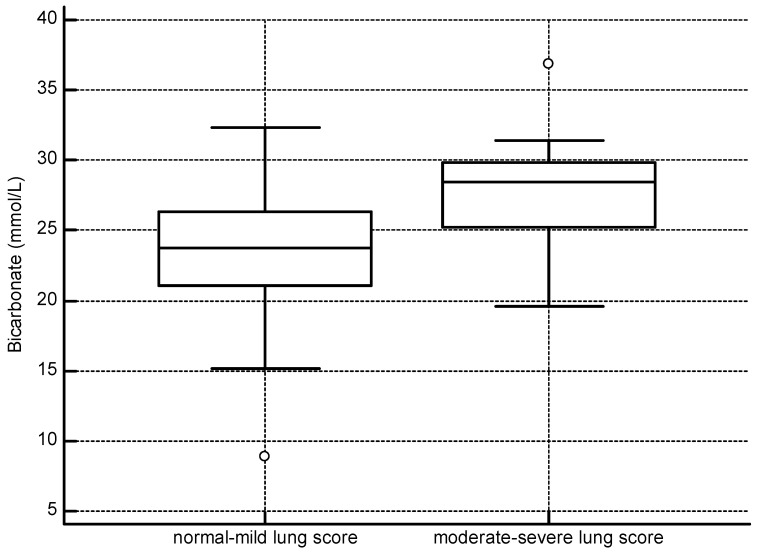
Barataria Bay dolphins with more severe pulmonary disease had higher bicarbonate concentrations (normal–mild lung score *n* = 40, mean = 23.6; moderate–severe lung score *n* = 19, mean = 27.7, *p* = 0.001).

Similar to the overall analysis, in BB dolphins with moderate–severe lung scores, base excess was more positive (*p* < 0.001), and HCO_3_ (*p* = 0.001), TCO_2_ (*p* = 0.011), and pH (*p* < 0.001) were higher than in dolphins with normal–mild lung scores. Differences in bicarbonate concentrations between the two lung score groups are depicted in Figure 4. The mean time between net set and blood gas sampling was 21 min, and no significant difference was found between lung score groups (*p* = 0.3563). No other factors significantly influenced the variables. See Table 3 for a summary of these findings. Twenty of fifty-one (39%) BB dolphins were assigned guarded to grave prognostic scores based on the complete clinical picture (eight BB animals did not have sufficient data for this assessment). In 1/4 (25%) failed and 2/3 (66%) successful BB pregnancies, mothers were assigned guarded to grave prognostic scores.

### 3.8. Time Differential Analysis

No significant difference was noted in the change in ETCO_2_, PCO_2_, TCO_2_, or lactate values over time in BB dolphins with moderate–severe lung scores (*n* = 18) compared to those with normal–mild lung scores (*n* = 24) (*p* = 0.469, Table 4).

### 3.9. Prognostic Score Analysis

The prognostic score for the BB cohort was not found to significantly correlate with ETCO_2_, PCO_2_, HCO_3_, base excess, or TCO_2_ values (*p* = 0.122).

## 4. Discussion

### 4.1. Pulmonary Disease

Nearly one-third of the bottlenose dolphins in Barataria Bay sampled for this study showed sonographic evidence of moderate to severe pulmonary disease six and seven years following the *Deepwater Horizon* (DWH) oil spill [2]. This is cause for significant concern, considering the persistence of these lesions and the fact that pneumonia is a principal cause of death in wild stranded dolphins [35,36,37]. The prevalence of moderate to severe pulmonary disease in this cohort is not statistically different from that found in BB dolphins in 2011 (34%, [2]), 2013, and 2014 (23% and 25%, respectively, [6]). It is, however, significantly higher than that reported for a non-oiled wild population in Sarasota Bay, Florida (7%, [6], *p* = 0.012). Not surprisingly, BB dolphin lung scores were more severe compared to those in dolphins managed by the Navy, despite intentional selection bias for Navy dolphins with respiratory disease. The Navy cohort not only did not experience the historic oil exposure, but they also benefit from daily health monitoring and comprehensive veterinary care, making them a valuable control group for the wild BB dolphins. 

Blood gas values differed between dolphins with normal–mild and those with moderate–severe lung disease, suggesting that the sonographic lung lesions observed in Barataria Bay dolphins have physiological impacts for these animals. Dolphins with moderate–severe lung scores had higher bicarbonate and TCO_2_ concentrations than dolphins with normal–mild scores. These trends were true for both the overall analysis and the BB cohort, since the BB cohort was the sole source for dolphins with moderate–severe lung scores. Few studies have reported reference ranges for carbon dioxide or bicarbonate concentrations in bottlenose dolphins, but in a population of managed dolphins, mean total carbon dioxide concentrations ranged from 23.3 to 23.9 mEq/L across age ranges and sexes [38]. Bicarbonate values in terrestrial animals can be estimated by subtracting 1.2 from total CO_2_ [39]. Considering these references, both lung score groups would be considered to have elevated bicarbonate and TCO_2_ concentrations, with more pronounced (moderate) elevations in dolphins with more severe lung scores.

The larger acid–base clinical picture can often shed light on the cause of elevated bicarbonate and TCO_2_ concentrations. For dolphins with moderate–severe lung disease, associated parameters (pH, PCO_2_, base excess, anion gap) were within the i-STAT normal range; however, considering the sampling conditions, these findings are not physiologically appropriate. An exercise-induced respiratory acidosis (indicated by higher TCO_2_ and PCO_2_ and lower pH values) followed by a metabolic acidosis (indicated by higher bicarbonate and lower pH) would be expected in those animals [40] due to the chase and capture scenario that occurred just prior to BB cohort sampling. Lower pH and higher PCO_2_ were observed within the normal–mild lung disease group (aligning with the expected exercise-induced respiratory acidosis), but not the moderate–severe group, possibly due to animals with more severe disease having a chronically altered respiratory physiology. With chronic respiratory disease, respiratory acidosis is expected to be compensated over time by metabolic alkalosis (hydrogen ion loss through renal excretion) to help moderate the blood pH level. However, with an acute exercise-induced respiratory acidosis, such as that expected with the BB cohort, metabolic compensation would not have time to establish. In humans, severe chronic pulmonary disease can predispose humans to exercise-induced hypercapnia due to mechanical ventilatory restrictions and ventilation–perfusion mismatch [41], which contradicts the pattern seen here in dolphins with more severe respiratory disease. It is possible that the more pronounced elevations in bicarbonate and TCO_2_ concentrations seen in the moderate–severe lung score dolphins are more suggestive of the chronic processes of primary respiratory acidosis and compensatory metabolic alkalosis, presumably related to the observed lung disease, than an acute exercise-induced hypercapnia. Thus, the dolphins with normal–mild lung scores demonstrated the expected response to the sampling conditions, whereas the dolphins with more severe lung disease did not. The pulmonary disease groups had no significant difference in the time between net set and blood draw, making a difference in exertion an unlikely explanation for the observed trends. It is also important to note that there are no previously published dolphin reference ranges for blood gas data and all the data in this study were interpreted relative to one another, and to general mammalian and i-STAT reference ranges. 

Alternative differential diagnoses for the observed elevated bicarbonate levels in dolphins with more severe lung disease include other mechanisms for hydrogen ion loss (vomiting, renal hydrogen ion loss due to diuretics, primary mineralocorticoid excess), intracellular shift of hydrogen ions, as would occur with hypokalemia, volume contraction, ingestion of an alkalotic agent, or other causes of metabolic alkalosis. While vomiting cannot be completely ruled out, evidence of recently ingested fish was observed on abdominal ultrasound in some animals. No evidence of mineralocorticoid excess was found in these dolphins; in fact, hypoadrenocorticism is a common finding in the Barataria Bay cohort [2,6]. Neither volume contraction nor hypokalemia were observed in BB dolphins [42]. Diuretics were not administered, and while ingestion of an alkalotic agent cannot be ruled out, there was no evidence of this. In this case, the association with lung disease would likely be a coincidence, or lung disease could be making these animals more susceptible to other conditions. The presence of metabolic acidosis (due to exercise-induced hyperlactatemia) in BB dolphins may complicate the acid–base interpretation; however, lactate did not significantly differ between dolphins with normal–mild and moderate–severe lung scores. Additional studies with more advanced pulmonary function testing are needed to further explore these potential trends.

The alveolar-interstitial syndrome observed sonographically in BB dolphins can be consistent with pneumonia, pulmonary edema, and/or pulmonary fibrosis [6], but the exact nature of the lesions cannot be identified based on the ultrasound alone. Only one-third of dolphins with sonographic evidence of disease also had outwards signs of pulmonary disease during their brief examination. The insensitivity of the physical exam for respiratory disease diagnosis is well-known in managed care dolphins, as dolphins often mask illness well and/or only show non-specific signs of illness, such as decreased appetite or lethargy. Despite their ability to cause impairments to respiratory function and health, certain sonographically observed lesions such as pulmonary fibrosis are also less likely to produce signs of respiratory disease upon physical exam. Human patients with pulmonary fibrosis have been shown to have restrictive ventilatory defects with more advanced pulmonary function tests [43], including decreased forced vital capacity on spirometry tests and decreased pulmonary diffusion [44,45]. Patients with diffuse parenchymal lung disease (such as pulmonary fibrosis) also have a high incidence of pulmonary hypertension, which is linked to exercise limitations and a poor prognosis in humans [46]. The observed differences in certain blood gas parameters between dolphins with normal–mild and moderate–severe lung scores suggest that there are likely physiological effects associated with the observed sonographic lesions, even when not associated with physical exam abnormalities. 

In the Navy dolphin with outward evidence of lung disease but no sonographic evidence, ongoing antimicrobial treatment, and the acute onset of pulmonary disease at the time of the initial ultrasound, may have played a role in this false-negative initial ultrasound report. Subsequent radiographs and ultrasound examination revealed mild to moderate lung disease five and seven days after the initial ultrasound, respectively, indicating that the lesions were either underdiagnosed during the initial ultrasound, or subsequently became detectable. Ultrasound is only capable of detecting diseases within superficial lung fields and is not a sensitive technique for diagnosing lesions obscured by the ribs or those deep in an aerated lung, which is another possible explanation for the initial ultrasound findings in this case. Computed tomography scans would also provide more sensitive tests for pulmonary disease, but this is not a practical tool for in-water field examinations on wild-caught dolphins. All BB dolphins with abnormal respiratory findings upon physical exam had at least mild lung lesions on the ultrasound. Pulmonary ultrasound is increasingly finding favor in certain circumstances within human medicine as well, further validating its clinical utility and continued use in dolphin field studies [47,48,49].

### 4.2. Barataria Bay vs. Navy Dolphin Cohorts

BB dolphins had higher PO_2_ values than Navy dolphins. This observed difference could be due to the slightly different blood draw techniques between populations, or possibly the increased arterial blood pressure due to the capture process (epinephrine release), making a higher contribution of arterial blood in the sample more likely for BB dolphins. BB dolphins had markedly higher lactate concentrations than Navy dolphins, which would be expected considering the former’s pre-sampling exercise. Ample precautions are taken to ensure the safety of the dolphins during the capture process and to minimize stress and chase time [26]. However, a temporary increase in lactate concentrations will inevitably result since the dolphins expend additional energy over a short time period, and thus are more likely to utilize anaerobic metabolism to meet their energetic needs. The Navy dolphins voluntarily presented their ventral flukes for blood draw and did not have an increase in energetic demands prior to doing so.

ETCO_2_ was also higher in the BB cohort, which could again reflect the increased energetic demands in BB dolphins prior to sampling and the resulting increased expiration of CO_2_ in their breath. The higher prevalence and severity of lung disease in the BB cohort could also be contributing to this trend, since elevated ETCO_2_ concentrations have been observed in a dolphin with pneumonia (van Elk et al. 2001) and dolphins with more severe lung scores tended to have higher ETCO_2_ values, although that difference was not significant. Adult Navy dolphins, however, were found to have lower ETCO_2_ than juveniles and subadults. The reasons behind this are unclear, but since ETCO_2_ can be largely affected by the respiratory rate, younger animals may be more prone to changes in respiratory rate, especially in response to novel procedures such as capnography, that could have driven the observed finding.

While there were inherent differences in the sampling procedures between Navy and BB dolphins, valuable insights were gained by having a control cohort with extensively documented medical histories. While the health assessments of wild captured dolphins are comprehensive, they provide only a snapshot of health, and some concurrent and prior conditions cannot be ruled out. The well-documented health histories of the Navy dolphins provided a clearer background upon which to interpret the results. By comparing BB dolphins to Navy dolphins, we were able to appreciate that between-cohort differences were likely due to anticipated physiological differences in the sampling methods, rather than undetected health conditions. These findings further support that certain parameters, especially ETCO_2_, may be useful indicators of the physiological state of dolphins, and they warrant further investigation. Building on the foundation laid here, a future study comparing a wild, non-oil-exposed cohort to an oil-exposed cohort would help to further differentiate the impacts of pulmonary disease from those of exercise- and/or stress-induced processes associated with capture.

### 4.3. Sex and Pregnancy

In the overall analysis, males had higher PCO_2_ values than females, but this trend was not observed in the separate cohort analyses. This finding could be due to differences in the respiratory rate [50], metabolic rate, pregnancy, or other physiologic factors. However, the respiratory rate did not differ between males and females in this study (*p* = 0.461), indicating that the other factors may be influencing this finding.

Pregnancy was not found to significantly influence any variables in this study, but this may be due to the low number of pregnant females, represented trimesters, or potential confounding factors in the study, such as the presence of pulmonary disease. Most pregnant females were in their first trimester, with just a few in their second trimester. Based on gestational physiology in other species, pregnant dolphins would be expected to have a mild chronic respiratory alkalosis, with a lower ETCO_2_, PCO_2_, and HCO_3_ (renal compensation), and higher SpO_2_, PO_2_, pH, and lactate than non-pregnant females [51]. Functional residual capacity and expiratory reserve volume have been found to significantly change in the third trimester of pregnancy in humans, which may explain the lack of a significant difference between pregnant and non-pregnant females in this study. Most of the changes expected in pregnancy also oppose the trends expected with pulmonary disease. There were insufficient numbers of dolphins with concurrent pregnancy and pulmonary disease in this study for meaningful comparisons with healthy pregnant females. This unfortunately limited our ability to explore the link between maternal pulmonary disease and reproductive failure. However, the data presented from this small number of pregnant females represent the first of their kind in the literature and provide a starting point from which additional insights into dolphin pregnancy acid–base physiology can be derived. Future work to control for confounding factors such as respiratory impairment and expanding the dataset to include more pregnant females in different trimesters will help to explore oxygenation in pregnant dolphins more thoroughly.

### 4.4. Capnography

As breath-holding diving mammals, bottlenose dolphins have an exceptionally large respiratory capacity and are capable of rapidly exchanging as much as 95% of their total lung capacity during a single breath [17,52,53]. For this reason, the ETCO_2_ values obtained by hovering the mask directly over the blowhole (vs. having a tight seal with the skin surrounding the blowhole) were likely negligibly affected by environmental air contamination. In fact, a small, controlled study showed no difference in ETCO_2_ values between these techniques (W. Musser, personal communication, May 2019). In free-ranging dolphin field studies, real-time diagnostics to evaluate oxygenation have been limited, and have recently involved the use of a pneumotachometer to obtain detailed information regarding pulmonary function, though not in pregnant females [53,54,55]. The clinical utility of non-invasive, handheld capnography for wild or managed dolphins had not previously been established.

Although significant relationships between ETCO_2_ and PCO_2_ were found in this study, the strength of the correlations is not sufficient for clinical utility. Only mild correlations were found between ETCO_2_ and PCO_2_ for dolphins with a mean PCO_2_–ETCO_2_ differential of +5.03 mmHg. A difference of +5 mmHg between these values is expected due to dead space in the airways diluting the alveolar PCO_2_. However, the range of the PCO_2_–ETCO_2_ differential was large (−23.0 to +23.3 mmHg) and the direction of the differential was unpredictable, complicating the clinical interpretation of ETCO_2_. These findings could have been driven, at least in part, by the sampling method being more representative of a mixed expired CO_2_ partial pressure vs. a true end-tidal CO_2_, which is more characteristic of a true alveolar sample. Respiratory disease can also increase the physiologic dead space in the lung, thus lowering exhaled CO_2_ relative to the PCO_2_ [56]. One study in a single dolphin noted an increase in ETCO_2_ with the onset of pneumonia [25], suggesting that a time series of capnography samples, including a healthy baseline sample, may be of more value than a single sample. Our findings show that while minimally invasive and easy to use in the field, a stand-alone capnograph sampling does not provide a clinically useful approximation for PCO_2_ or evaluation of respiratory disease in awake dolphins.

### 4.5. Blood Gas Interpretation in Dolphins

The i-STAT analyzer has been used in field studies with wild delphinids to measure blood gas, electrolytes, and other hematologic parameters [2,16,57,58]. Dolphin peripheral arteries are surrounded by veins in structures called periarterial venous retia, making it infeasible to reliably obtain pure arterial samples in awake animals [59], as is the gold standard for blood gas analysis. Interpreting blood gas data derived from a mixed venous–arterial sample is not ideal, since the value of certain parameters differs between arterial and venous blood (i.e., PO_2_ is lower and PCO_2_ is higher in venous blood). An undetermined amount of venous–arterial mixing, as was the case in the samples presented here, can limit and/or confound blood gas interpretation.

However, in recent years, human and veterinary clinicians have been increasingly using venous blood gas samples in certain situations. Kim et al. [60] found that venous pH, PCO_2_, HCO_3_, and TCO_2_ were suitable alternatives to their arterial equivalents when arterial blood was unobtainable in critically ill people. In a systematic review and meta-analysis study, Bloom et al. [61] reported that venous and arterial bicarbonate and pH had reasonable agreement. In small animals, blood gas values derived from a variety of non-arterial samples were found useful in evaluating the acid–base status [13,62]. In dolphins, Ridgway and McCormick [19] reported that pH, PO_2_, and PCO_2_ recorded from the central artery of the fluke’s periarterial venous rete were identical to those taken from the carotid artery during anesthesia. While interpretation may be more challenging, the literature shows that certain variables may be useful for evaluating pulmonary gas-exchange abnormalities, even in mixed venous–arterial samples. The degree of arterial/venous mixing may affect certain parameters, especially PO_2_ and PCO_2_, and this must be considered when interpreting the data. However, the detected differences in HCO_3_ and pH in dolphins with more severe pulmonary disease correspond with anticipated pathophysiological changes, and thus indicate that these parameters may be useful despite the challenges associated with blood gas sampling in dolphins.

The factors that dictate blood gas values in mammalian species are often multifactorial and complex, with six different causes of hypoxemia variably contributing to the arterial O_2_ and CO_2_ tension in any given patient, as follows: inspiratory hypoxia, hypoventilation, ventilation/perfusion mismatch, diffusion limitation, shunting, and reduced mixed venous oxygenation. Blood gas values are further affected by the body’s ability to compensate for gas-exchange disturbances [14]. While not ideal, this study indicated that even mixed arterial–venous blood gases may be clinically useful in evaluating dolphin lung disease.

## 5. Conclusions

The present study demonstrated that chronic respiratory acidemia with compensatory metabolic alkalosis may be present in dolphins with moderate–severe respiratory disease in Barataria Bay, LA. Although direct oxygenation measurements, such as pulse oximetry and partial pressure of O_2_ in blood, are difficult to obtain due to the dolphin anatomy and physiology, indirect measures such as bicarbonate and TCO_2_ concentrations show promise in evaluating the oxygenation status in dolphins. While easy to use in the field, the findings of this study do not strongly support single sampling with hand-held capnography as a clinically effective approximation for the partial pressure of CO_2_ in blood. Future studies with more advanced pulmonary function tests could help clarify the relationships between pulmonary function and pulmonary disease in oil-exposed dolphins. In order to better understand whether maternal hypoxia may affect fetal and perinatal health and survival, expanded studies to track pregnant dolphins for successful calving and to see how these parameters correlate to their eventual reproductive success or failure are needed.

## Figures and Tables

**Table 1:** Summary of samples (*n*) collected from wild-caught bottlenose dolphins (*Tursiops truncatus*) in Barataria Bay, LA (BB), and managed care *T. truncatus* in San Diego, CA (Navy). Age/length classes: juvenile (0–5 years OR ≤ 225 cm), subadult (>5 and <10 years OR >225 and <240 cm), adult (≥10 years OR ≥ 240 cm).

		BB	Navy	Total
Sex	Male	25	14	39
	Female	34	16	50
Age/Length Class	Juvenile	33	8	41
	Subadult	13	4	17
	Adult	13	18	31
Pregnancy	Pregnant Female	10	5	15
	Non-Pregnant Female	24	11	35
Ultrasound Lung Score	Normal–Mild	40	30	70
	Moderate–Severe	19	0	19

**Table 2:** Summary of pulmonary ultrasound scores (*n*) for managed (Navy) and wild (BB) dolphins. Pulmonary ultrasounds were scored as follows: normal (no evidence of disease), mild disease, moderate disease, or severe disease.

Pulmonary Ultrasound Scores	Normal	Mild	Moderate	Severe
Navy	25	5	0	0
BB	4	36	16	3

**Table 3:** Descriptive statistics for all dolphins, by cohort, and by lung score category. Parametric data are presented as mean (*n*, SD), and non-parametric data as median (*n*, range). * Denotes *p* < 0.05 for cohort, and # denotes *p* < 0.05 for lung score. For multiple comparisons among non-parametric data, the Holm’s Sequential Bonferroni correction was applied to evaluate for significance.

		Cohort		BB by Lung Score Category	
	All Dolphins	Navy (All Normal–Mild Lung Score)	BB	BB Normal–Mild Lung Score	BB Moderate–Severe Lung Score
Base Excess (mEq/L)	−1.0	−0.6	−2.0	−3.8 #	1.8 #
	(−24–12)	(3.2)	(6.0)	(5.7)	(4.7)
	*n* = 89	*n* = 30	*n* = 59	*n* = 40	*n* = 19
Bicarbonate (mEq/L)	25.6	26.0	24.9	23.6 #	27.7 #
	(8.9–36.9)	(2.9)	(4.7)	(4.5)	(3.8)
	*n* = 89	*n* = 30	*n* = 59	*n* = 40	*n* = 19
TCO_2_ (mmol/L)	27.0	27.6	26.3	25.2 #	28.6 #
	(10–39)	(3.0)	(4.9)	(4.6)	(4.8)
	*n* = 89	*n* = 30	*n* = 59	*n* = 40	*n* = 19
Lactate (mmol/L)	4.0	1.3 *	7.3 *	8.1	5.0
	(0.3–20.0)	(0.7)	(1.2–20.0)	(1.7–20)	(1.2–12.7)
	*n* = 89	*n* = 30	*n* = 59	*n* = 40	*n* = 19
PCO_2_ (mmHg)	53.4	53.8	53.3	54.1	51.4
	(5.7)	(5.2)	(6.0)	(5.8)	(6.2)
	*n* = 89	*n* = 30	*n* = 59	*n* = 40	*n* = 19
PO_2_ (mmHg)	62.8	51.4 *	68.0 *	68.0	68.0
	(18.0)	(18.1)	(46–116)	(47.0–116.0)	(46.0–97.0)
	*n* = 88	*n* = 30	*n* = 58	*n* = 39	*n* = 19
pH	7.294	7.289	7.279	7.243 #	7.333 #
	(6.903–7.456)	(0.037)	(6.903–7.456)	(6.903–7.393)	(7.162–7.456)
	*n* = 89	*n* = 30	*n* = 59	*n* = 40	*n* = 19
ETCO_2_ (mmHg)	46.8	40.6 *	51.9 *	52.0	51.6
	(31.2–75.2)	(33.4–65.4)	(9.7)	(9.2)	(10.9)
	*n* = 83	*n* = 30	*n* = 53	*n* = 36	*n* = 17

**Table 4:** Mean change in select values over time for BB dolphins by lung score category.

	Lung Score	
	Normal–Mild (*n* = 24)	Moderate–Severe (*n* = 18)
Δ ETCO_2_	3.9	2.1
Δ PCO_2_	0.9	0
Δ HCO_3_	3.7	1.6
Δ Lactate	−4.2	−2.2

## Data Availability

Data are publicly available through the Gulf of Mexico Research Initiative Information and Data Cooperative (GRIIDC), at: https://data.gulfresearchinitiative.org ((accessed on 19 April 2023) doi: 10.7266/N7H41PTV, 10.7266/n7-76aj-rp39, 10.7266/n7-63bk-a322, 10.7266/F4HGMXD5, and 10.7266/n7-0vt6-ax64).

## References

[1] United States of America, BP Exploration & Production, Inc. (2015). Findings of fact and conclusions of law: Phase two trial. Re: Oil Spill by the Oil Rig Deepwater Horizons in the Gulf of Mexico, on 20 April 2010.

[2] Schwacke L.H., Smith C.R., Townsend F.I., Wells R.S., Hart L.B., Balmer B.C., Collier T.K., De Guise S., Fry M.M., Guillette J.L.J. (2014). Health of common bottlenose dolphins (*Tursiops truncatus*) in Barataria Bay, Louisiana, following the *Deepwater Horizon* oil spill. Environ. Sci. Technol..

[3] Lane S.M., Smith C.R., Mitchell J., Balmer B.C., Barry K.P., McDonald T., Mori C.S., Rosel P.E., Rowles T.K., Speakman T.R. (2015). Reproductive outcome and survival of common bottlenose dolphins sampled in Barataria Bay, Louisiana, USA, following the Deepwater Horizon oil spill. Proc. Biol. Sci..

[4] Venn-Watson S., Colegrove K.M., Litz J., Kinsel M., Terio K., Saliki J., Fire S., Carmichael R., Chevis C., Hatchett W. (2015). gland and lung lesions in Gulf of Mexico common bottlenose dolphins (*Tursiops truncatus*) found dead following the *Deepwater Horizon* oil spill. PLoS ONE.

[5] Kellar N.M., Speakman T.R., Smith C.R., Lane S.M., Balmer B.C., Trego M.L., Catelani K.N., Robbins M.N., Allen C.D., Wells R.S. (2017). Low reproductive success rates of common bottlenose dolphins *Tursiops truncatus* in the northern Gulf of Mexico following the *Deepwater Horizon* disaster (2010–2015). Endanger. Species Res..

[6] Smith C.R., Rowles T.K., Hart L.B., Townsend F.I., Wells R.S., Zolman E.S., Balmer B.C., Quigley B., Ivancic M., McKercher W. (2017). Slow recovery of Barataria Bay dolphin health following the *Deepwater Horizon* oil spill (2013–2014), with evidence of persistent lung disease and impaired stress response. Endanger. Species Res..

[7] Colegrove K., Venn-Watson S., Litz J., Kinsel M., Terio K., Fougeres E., Ewing R., Pabst D., McLellan W., Raverty S. (2016). Fetal distress and in utero pneumonia in perinatal dolphins during the Northern Gulf of Mexico unusual mortality event. Dis. Aquat. Org..

[8] Liu Y.Z., Roy-Engel A.M., Baddoo M.C., Flemington E.K., Wang G., Wang H. (2016). The impact of oil spill to lung health—Insights from an RNA-seq study of human airway epithelial cells. Gene.

[9] Nie H., Liu H., Shi Y., Lai W., Liu X., Xi Z., Lin B. (2022). Combined multi-omics analysis reveals oil mist particulate matter-induced lung injury in rats: Pathological damage, proteomics, metabolic disturbances, and lung dysbiosis. Ecotox. Environ. Safe..

[10] Tuder R.M., Yun J.H., Bhunia A., Fijalkowska I. (2007). Hypoxia and chronic lung disease. J. Mol. Med..

[11] Hutter D., Kingdom J., Jaeggi E. (2010). Causes and Mechanisms of Intrauterine Hypoxia and its Impact on the Fetal Cardiovascular System: A Review. Int. J. Pediatr..

[12] Getahun D., Ananth C.V., Oyelese Y., Peltier M.R., Smulian J.C., Vintzileos A.M. (2007). Acute and chronic respiratory disease in pregnancy: Associations with spontaneous premature rupture of membranes. J. Matern. Fetal Neonatal Med..

[13] Ilkiw J.E., Rose R.J., Martin I.C.A. (1991). A Comparison of Simultaneously Collected Arterial, Mixed Venous, Jugular Venous and Cephalic Venous Blood Samples in the Assessment of Blood-Gas and Acid-Base Status in the Dog. J. Vet. Intern. Med..

[14] Wagner P.D. (2015). The physiological basis of pulmonary gas exchange: Implications for clinical interpretation of arterial blood gases. Eur. Respir. J..

[15] Trulock E.P., Walker K.W., Hall W.D., Hurst J.W. (1990). Arterial Blood Gases. Clinical Methods: The History, Physical and Laboratory Examinations.

[16] Shaffer S.A., Costa D.P., Williams T.M., Ridgway S.H. (1997). Diving and swimming performance of white whales, *Delphinapterus leucas*: An assessment of plasma lactate and blood gas levels and respiratory rates. J. Exp. Biol..

[17] Williams T.M., Haun J.E., Friedl W.A. (1999). The diving physiology of bottlenose dolphins (*Tursiops truncatus*). I. Balancing the demands of exercise for energy conservation at depth. J. Exp. Biol..

[18] Haulena M., Heath R.B., Dierauf L.A., Gulland F.M.D. (2001). Marine Mammal Anesthesia. CRC Handbook of Marine Mammal Medicine.

[19] Ridgway S.H., McCormick J.G. (1967). Anesthetization of Porpoises for Major Surgery. Science.

[20] Rieu M., Gautheron B. (1968). Preliminary observations concerning a method for introduction of a tube for anesthesia in small delphinids. Life Sci..

[21] ACVAA (American College of Veterinary Anesthesia and Analgesia) (2009). Recommendations for Monitoring Anesthetized Veterinary Patients. http://www.acvaa.org/docs/Small_Animal_Monitoring_2009.doc.

[22] Dold C., Ridgway S., West G.W., Heard D., Caukett N. (2007). Cetaceans. Zoo Animal and Wildlife Immobilization and Anesthesia.

[23] Kelmer E., Scanson L.C., Reed A., Love L.C. (2009). Agreement between values for arterial and end-tidal partial pressure of carbon dioxide in spontaneously breathing, critically ill dogs. J. Am. Vet. Med. Assoc..

[24] Mortola J.P., Seguin J. (2009). End-tidal CO2 in some aquatic mammals of large size. Zoology.

[25] van Elk C.E., Epping N., Gans S.L.M. (2001). Pulmonary function measurements in dolphins using capnography. Vet. Rec..

[26] Wells R.S., Rhinehart H.L., Hansen L.J., Sweeney J.C., Townsend F.I., Stone R., Casper D., Scott M.D., Hohn A.A., Rowles T.K. (2004). Bottlenose dolphins as marine ecosystem sentinels: Developing a health monitoring system. EcoHealth.

[27] Barratclough A., Wells R.S., Schwacke L.H., Rowles T.K., Gomez F.M., Fauquier D.A., Sweeney J.C., Townsend F.I., Hansen L.J., Zolman E.S. (2019). Health Assessments of Common Bottlenose Dolphins (*Tursiops truncatus*): Past, Present, and Potential Conservation Applications. Front. Vet. Sci..

[28] McFee W.E., Schwacke J.H., Stolen M.K., Mullin K.D., Schwacke L.H. (2010). Investigation of growth phases for bottlenose dolphins using a Bayesian modeling approach. Mar. Mammal Sci..

[29] Smith C.R., Solano M., Lutmerding B.A., Johnson S.P., Meegan J.M., Le-Bert C.R., Emory-Gomez F., Cassle S., Carlin K., Jensen E.D. (2012). Pulmonary ultrasound findings in a bottlenose dolphin *Tursiops truncatus* population. Dis. Aquat. Org..

[30] Wells R., Smith C.R., Sweeney J., Townsend F.I., Fauquier D., Stone R., Langan J., Schwacke L., Rowles T.K. (2014). Fetal survival for bottlenose dolphins, Tursiops truncatus, in Sarasota Bay, Florida. Aquat. Mamm..

[31] Everitt B.S., Dunn G. (1991). Applied Multivariate Data Analysis.

[32] Holm S. (1979). A simple sequentially rejective multiple test procedure. Scand. J. Stat..

[33] Abdi H., Salkind N. (2010). Holm’s Sequential Bonferroni Procedure. Encyclopedia of Research Design.

[34] Bland J.M., Altman D.G. (1986). Statistical methods for assessing agreement between two methods of clinical measurement. Lancet.

[35] Di Guardo G., Agrimi U., Morelli L., Cardeti G., Terracciano G., Kennedy S. (1995). Post mortem investigations on cetaceans found stranded on the coasts of Italy between 1990 and 1993. Vet. Rec..

[36] Cornaglia E., Rebora L., Gilli C., Di Guardo G. (2000). Histopathological and immunohistochemical studies on cetaceans found stranded on the coast of Italy between 1990 and 1997. J. Vet. Med. A Physiol. Pathol. Clin. Med..

[37] Bogomolni A.L., Pugliares K.R., Sharp S.M., Patchett K., Harry C.T., LaRocque J.M., Touhey K.M., Moore M. (2010). Mortality trends of stranded marine mammals on Cape Cod and southeastern Massachusetts, USA, 2000 to 2006. Dis. Aquat. Organ..

[38] Venn-Watson S., Jensen E.D., Ridgway S.H. (2007). Effects of age and sex on clinicopathologic reference ranges in a healthy managed Atlantic bottlenose dolphin population. J. Am. Vet. Med Assoc..

[39] Coles E.H. (1986). Veterinary Clinical Pathology.

[40] Stringer W., Casaburi R., Wasserman K. (1992). Acid-base regulation during exercise and recovery in humans. J. Appl. Physiol..

[41] O’Donnell D.E., D’Arsigny C., Fitzpatrick M., Webb K.A. (2002). Exercise hypercapnia in advanced chronic obstructive pulmonary disease: The role of lung hyperinflation. Am. J. Respir. Crit. Care Med..

[42] Schwacke L.H., Marques T.A., Thomas L., Booth C.G., Balmer B.C., Barratclough A., Colegrove K., De Guise S., Garrison L.P., Gomez F.M. (2022). Modeling population effects of the Deepwater Horizon oil spill on a long-lived species. Conserv. Biol..

[43] Bennett D., Mazzei M.A., Squitieri N.C., Bargagli E., Refini R.M., Fossi A., Volterrani L., Rottoli P. (2017). Familial pulmonary fibrosis: Clinical and radiologic characteristics and progression analysis in different high resolution-CT patterns. Respir. Med..

[44] Lapinsky S.E., Tram C., Mehta S., Maxwell C.V. (2014). Restrictive lung disease in pregnancy. Chest.

[45] Schmidt S.L., Tayob N., Han M.K., Zappala C., Kervitsky D., Murray S., Wells A.U., Brown K.K., Martinez F.J., Flaherty K.R. (2014). Predicting Pulmonary Fibrosis Disease Course from Past Trends in Pulmonary Function. Chest.

[46] Seeger W., Adir Y., Barberà J.A., Champion H., Coghlan J.G., Cottin V., De Marco T., Galiè N., Ghio S., Gibbs S. (2013). Pulmonary hypertension in chronic lung diseases. J. Am. Coll. Cardiol..

[47] Koenig S., Chandra S., Alaverdian A., Dibello C., Mayo P.H., Narasimhan M. (2014). Ultrasound Assessment of Pulmonary Embolism in Patients Receiving CT Pulmonary Angiography. Chest.

[48] Bass C.M., Sajed D.R., Adedipe A.A., West T.E. (2015). Pulmonary ultrasound and pulse oximetry versus chest radiography and arterial blood gas analysis for the diagnosis of acute respiratory distress syndrome: A pilot study. Crit. Care.

[49] Irwin Z., Cook J.O. (2016). Advances in Point-of-Care Thoracic Ultrasound. Emerg. Med. Clin. N. Am..

[50] Terasawa F., Ohizumi H., Ohshita I. (2010). Effect of Breath-Hold on Blood Gas Analysis in Captive Pacific White-Sided Dolphins (*Lagenorhynchus obliquidens*). J. Vet. Med. Sci..

[51] McAuliffe F., Kametas N., Costello J., Rafferty G.F., Greenough A., Nicolaides K. (2002). Respiratory function in singleton and twin pregnancy. BJOG.

[52] Ridgway S.H., Scronce B.L., Kanwisher J. (1969). Respiration and Deep Diving in the Bottlenose Porpoise. Science.

[53] Fahlman A., Loring S.H., Levine G., Rocho-Levine J., Austin T., Brodsky M. (2015). Lung mechanics and pulmonary function testing in cetaceans. J. Exp. Biol..

[54] Askenov A.A., Yeates L., Pasamontes A., Siebe C., Zrodnikov Y., Simmons J., McCartney M.M., Deplanque J.P., Wells R.S., Davies C.E. (2014). Metabolite Content Profiling of Bottlenose Dolphin Exhaled Breath. Anal. Chem..

[55] Yeates L.C., Carlin K.P., Baird M., Venn-Watson S., Ridgway S. (2014). Nitric oxide in the breath of bottlenose dolphins: Effects of breath hold duration, feeding and lung disease. Mar. Mammal Sci..

[56] Kiefmann M., Tank S., Tritt M.O., Keller P., Heckel K., Schulte-Uentrop L., Olotu C., Schrepfer S., Goetz A.E., Kiefmann R. (2019). Dead space ventilation promotes alveolar hypocapnia reducing surfactant secretion by altering mitochondrial function. Thorax.

[57] Varela R.A., Schwacke L., Fair P.A., Bossart G.D. (2006). Effects of duration of capture and sample handling on critical care blood analytes in free-ranging bottlenose dolphins. J. Am. Vet. Med. Assoc..

[58] Sharp S.M., Knoll J.S., Moore M.J., Moore K.M., Harry C.T., Hoppe J.M., Niemeyer M.E., Robinson I., Rose K.S., Sharp W.B. (2014). Hematological, biochemical, and morphological parameters as prognostic indicators for stranded common dolphins (*Delphinus delphis*) from Cape Cod, Massachusetts, U.S.A.. Mar. Mammal Sci..

[59] Bossart G.D., Reidarson T.H., Dierauf L.A., Duffield D.A., Dierauf L.A., Gulland F.M.D. (2001). Clinical pathology. CRC Handbook of Marine Mammal Medicine.

[60] Kim B.R., Park S.J., Shin H.S., Jung Y.S., Rim H. (2013). Correlation between peripheral venous and arterial blood gas measurements in patients admitted to the intensive care unit: A single center study. Kidney Res. Clin. Pract..

[61] Bloom B.M., Grundlingh J., Bestwick J., Harris T. (2014). The role of venous blood gas in the Emergency Department: A systematic review and meta-analysis. Eur. J. Emerg. Med..

[62] Bachmann K., Kutter A.P., Schefer R.J., Marly-Voquer C., Sigrist N. (2017). Determination of reference intervals and comparison of venous blood gas parameters using standard and non-standard collection methods in 24 cats. J. Feline Med. Surg..

